# Associations between rates of unassisted inpatient falls and levels of registered and non-registered nurse staffing

**DOI:** 10.1093/intqhc/mzt080

**Published:** 2013-11-13

**Authors:** Vincent S. Staggs, Nancy Dunton

**Affiliations:** 1 Department of Biostatistics, University of Kansas Medical Center, 3901 Rainbow Boulevard, MS 3060, Kansas City, KS 66160, USA; 2 School of Nursing University of Kansas Medical Center, 3901 Rainbow Boulevard, MS 3060, Kansas City, KS 66160, USA

**Keywords:** patient safety, accidental falls, nursing personnel, personnel staffing and scheduling

## Abstract

**Objective:**

To enhance understanding of how nurse staffing relates to unassisted falls by exploring non-linear associations between unassisted fall rates and levels of registered nurse (RN) and non-RN staffing on 5 nursing unit types, thereby enabling managers to improve patient safety by making better-informed decisions about staffing.

**Design:**

Cross-sectional analysis of routinely collected data using hierarchical negative binomial regression.

**Settings:**

8069 nursing units in 1361 U.S. hospitals participating in the National Database of Nursing Quality Indicators^®^.

**Main outcome measure:**

Rate of unassisted falls per inpatient day.

**Results:**

Associations between unassisted fall rates and nurse staffing varied by unit type. For medical–surgical units, higher RN staffing was weakly associated with lower fall rates. On step-down and medical units, the association between RN staffing and fall rates depended on the level of staffing: At lower staffing levels, the fall rate increased as staffing increased, but at moderate and high staffing levels, the fall rate decreased as staffing increased. Higher levels of non-RN staffing were generally associated with higher fall rates..

**Conclusions:**

Increasing non-RN staffing seems ineffective at preventing unassisted falls. Increasing RN staffing may be effective, depending on the unit type and the current level of staffing.

## Introduction

The topic of inpatient falls has received a great deal of attention from researchers, hospitals, patient safety organizations and government agencies. Falls are costly, both in dollars [1] and in human suffering, primarily because they cause injuries. Despite evidence that an injury is more likely in an unassisted fall [2], the distinction between assisted and unassisted falls has not been considered in most research on falls, nor have unassisted falls been the focus of patient safety initiatives. Our limited understanding of the relation between fall rates and staffing levels of registered and non-registered nurses (RN and non-RN) leaves hospital managers concerned about fall-related injuries underinformed regarding what kind of staffing changes might be beneficial on various unit types.

### Nurse staffing and falls

The importance of nurse staffing to patient safety has been of particular interest to researchers since the 1990s. Higher levels of RN staffing and RN skill mix (the proportion of nursing care hours provided by RNs) have been linked to lower rates of several adverse patient events [3–5], but studies of the effects of nurse staffing and skill mix on falls have yielded mixed results [6–8]. Most studies of the association between patient outcomes and staffing or skill mix have been limited by the assumption that the association is linear, which may be one reason results have been inconsistent in the case of falls [3]. Indeed, there is evidence that the associations between nurse staffing and the rates of total and injury falls are non-linear, and that these associations vary by nursing unit type [9].

Examining the association between nurse staffing and falls under the assumption that it is linear can lead to one of three basic findings: higher staffing is associated with lower fall rates, higher staffing is associated with higher fall rates or there is insufficient evidence to conclude that staffing and fall rates are associated. One of the limitations of this approach is that none of these findings provides much guidance to managers in setting appropriate staffing levels. For example, the implication of the first finding is that ‘more is better’ when it comes to nurse staffing, but this gives no indication of how much staffing is enough or of how much might be too much.

In a rare study of unassisted falls [10], researchers fit a quadratic model to data for 6 unit types and found that the unassisted fall rate increased with total nurse staffing through ∼9 nursing hours per patient day (HPPD) and then began to drop. The conclusions that can be drawn from this finding are limited by the model used in the study, which did not allow for different staffing-fall rate associations on different types of nursing units, but the finding is important as the first published evidence of a non-linear association between nurse staffing and the unassisted fall rate.

Complicating research on the association between staffing and falls is the fact that not all nursing care hours are equivalent because not all nursing personnel have the same education, training and experience. Researchers in one study [11] found that nursing units with more experienced RNs had lower total fall rates, and Staggs, Knight and Dunton [10] reported that units with longer average RN tenure on the unit tended to have lower unassisted fall rates. In a study of 2004 data from 5388 units in 636 hospitals participating in the National Database of Nursing Quality Indicators^®^ (NDNQI^®^), Lake, Shang, Klaus and Dunton [8] found that higher RN staffing levels were associated with lower total fall rates in intensive care units (ICUs), whereas higher staffing levels of both licensed practical nurses (LPNs) and nursing assistants were associated with higher fall rates in non-ICUs. It is worth noting that had Lake *et al.* considered only total nurse staffing, the combination of the effects of RN, LPN and nursing assistant staffing might have produced a null finding for the effect of total staffing, and failure to differentiate among types of nursing care hours may be another reason researchers have not consistently found a significant association between staffing and fall rates.

In summary, the association between staffing and falls in general is unclear in spite of the numerous studies devoted to the topic, and even less is known about the association between staffing and unassisted falls. Research involving non-linear models of these associations may be crucial to understanding how and why staffing affects patient falls in general, and unassisted falls in particular.

### Purpose

The purpose of this study was to explore non-linear associations between unassisted fall rates and levels of RN and non-RN staffing. Separate models were fit for each of 5 nursing unit types, allowing both the average fall rate and the association between staffing and the fall rate to vary by unit type.

## Methods

### Sample and data

We extracted monthly unit-level data on nurse staffing and inpatient falls for 2011 from the NDNQI. The NDNQI collects data on nursing-related measures from over 1900 U.S. hospitals (about one-third of all U.S. hospitals). Neither the study sample nor the larger set of NDNQI hospitals is a random sample of hospitals; participation in NDNQI is voluntary, and not all participating hospitals choose to submit data for all eligible nursing units.

We limited the sample to units in general hospitals (facilities primarily providing acute care for medical–surgical patients) and rehabilitation hospitals (facilities offering intensive rehabilitation services following acute care hospitalization) for which data on staffing and falls were available. Unit-months with fewer than 150 patient days or missing data on staffing or falls were excluded. The final sample comprised 1557 step-down, 2010 medical, 2567 medical–surgical, 1395 surgical, and 540 rehabilitation units in 1332 general hospitals and 29 rehabilitation hospitals. There was some diversity among the sample hospitals; 617 were teaching facilities (clinical sites for medical interns or residents), and 984 were smaller facilities with fewer than 300 staffed beds.

There were 87 544 unit-months of data in the final data set (10.8 months of non-missing data per unit, on average). All 12 months of data were available for 6176 (76.5%) of the 8069 units in the final data set.

### Variables

The dependent measure was the number of unassisted falls per patient day as reported monthly for each unit. For descriptive purposes, we computed overall rates of unassisted falls per 1000 patient days and percentages of falls unassisted. NDNQI defines an assisted fall as an unplanned descent to the floor in which a member of the hospital staff attempts to ease the patient down or otherwise break the fall to minimize its impact.

We considered two unit-level staffing variables and two hospital-level control variables as predictors of the unassisted fall rate. At the unit level, RN HPPD was defined as the sum of nursing care hours provided by RNs during the month divided by the sum of the unit's patient days for the month. Non-RN HPPD was defined in the same way using nursing care hours provided by LPNs and assistive personnel. As defined by NDNQI, nursing care hours include only productive hours provided by nursing employees who are assigned to a specific unit and spend more than half their shift in direct patient care. At the hospital level, two dichotomous variables were used to classify each hospital by teaching status (teaching or non-teaching) and bed size (<300 or ≥300 staffed beds).

### Statistical analysis

The research design was cross sectional. We analyzed the associations between staffing and unassisted fall rates using three-level generalized linear mixed models [12]. The unit-month was the basic unit of analysis. We accounted for the hierarchical structure of the data by including a random hospital intercept (to account for nesting of units within hospitals) and a random unit intercept (to account for correlation among a unit's repeated measures) in each model. The monthly count of falls was assumed to follow a negative binomial distribution, which is commonly used for modeling count data [13]. By including patient days for the month as an exposure variable in the model, we modeled the unassisted fall rate as the dependent measure.

We used restricted cubic splines [14] to model non-linear associations between the unassisted fall rate and the two staffing variables. The cubic spline model is a well-established tool for fitting a curve to capture a highly non-linear association between a predictor and response variable. Because this method may not be familiar to readers, some explanation follows.

Ordinary linear regression is used to find a straight line that best fits the data. This can be visualized as positioning a pencil through the scatterplot of data. With a regression spline, two or more regression line segments are fit to the data. The line segments are joined at one or more points (called ‘knots’), and as in ordinary regression, the intercept and slope of each line segment is chosen to achieve the best fit to the data. In visual terms, the researcher places two or more pencils, end to end, through the scatterplot.

In a cubic spline model, the straight regression line segments of a regression spline are replaced by cubic polynomials. Each polynomial is a curve that can change directions up to two times to fit the data. Like the line segments in a regression spline, these curves are joined at the knots, and the overall result is a smooth curve. Fitting a cubic spline model is like fitting segments of flexible string through the scatterplot instead of pencils.

The cubic spline models we fit were restricted, meaning that we fit cubic polynomials in between consecutive knots and straight line segments below the first knot and above the last knot, as recommended by Harrell [14]. We selected the 5th, 25th, 50th, 75th and 95th percentile of each staffing variable as knots for the spline.

Modeling was carried out using the GLIMMIX Procedure in SAS 9.2 (SAS Institute Inc., Cary, NC, USA). We included hospital bed size and teaching status as control variables in all models. For each unit type, the first two models fit were (i) a spline model for RN HPPD with non-RN HPPD included as a linear predictor and (ii) a spline model for non-RN HPPD with RN HPPD included as a linear predictor.

For each spline model, we computed a contrast to test the null hypothesis that the non-linear spline coefficients equal zero. Rejection of this hypothesis indicates that a linear model is inadequate. If this linearity hypothesis was rejected (at *α* = 0.05) for exactly one of the two spline models (RN HPPD and non-RN HPPD) for a unit type, we selected that spline model as the final model. If neither spline contrast was significant, we fit a third model with both staffing variables included as linear predictors and selected this linear model as the final model. The case of both spline contrasts being significant did not arise.

We computed the squared correlation between the observed and model-predicted fall counts as a measure of each final model's predictive power. This pseudo-*R*^2^ value is an estimate of the expected proportion of variation in the fall counts accounted for by the predictors [15].

## Results

There were 203 094 patient falls reported during the 57 518 290 patient days in the study (3.53 falls per 1000 patient days). Of these falls, 171 792 (84.6%) were unassisted, and 27 167 (13.4%) were assisted, the remainder being unclassified. Descriptive information is given in Table 1.

**Table 1:** Descriptive information by unit type

Unit type	Units	Unit-months	RN HPPD^a^	Non-RN HPPD^b^	Unassisted fall rate^c^	Percent unassisted^d^
Step-down	1557	16 862	7.4	2.7	2.7	86.2
Medical	2010	21 145	5.8	2.8	3.3	88.4
Medical–surgical	2567	27 649	5.8	2.8	3.0	87.2
Surgical	1395	15 231	6.0	2.8	2.2	83.9
Rehabilitation	540	5657	4.5	3.5	4.9	80.2

^a^RN HPPD = total RN hours divided by total patient days.

^b^Non-RN HPPD = total non-RN hours divided by total patient days.

^c^Unassisted falls reported per 1000 patient days.

^d^Percent of falls reported as unassisted.

Non-RN HPPD did not have a significant non-linear association with the unassisted fall rate for any unit type. The RN HPPD spline contrast was significant only for step-down (*P*-value = 0.040) and medical units (*P*-value = 0.017). Because neither spline contrast was significant for medical–surgical, surgical and rehabilitation units, we selected the linear model as the final model for these three unit types. Results from the final model for each unit type are provided in Table 2. Pseudo-*R*^2^ values ranged from 0.32 to 0.50, corresponding to correlations between the observed and model-predicted fall counts ranging from 0.57 to 0.71.

**Table 2:** Regression model results by unit type

Unit type	Variable	Exp(*β*)	95% CI for exp(*β*)	*Z*	*P*-value
Step-down (pseudo-*R*^2^ = 0.38)	Teaching status^a^	1.05	0.99, 1.11	1.49	0.136
	Bed size^b^	0.93	0.88, 0.99	−2.24	0.025
	Non-RN HPPD^c^	1.03	1.01, 1.05	3.53	<0.001
Medical (pseudo-*R*^2^ = 0.36)	Teaching status	1.04	0.99, 1.10	1.48	0.138
	Bed size	0.96	0.91, 1.01	−1.62	0.106
	Non-RN HPPD	1.04	1.02, 1.06	4.47	<0.001
Medical–surgical (pseudo-*R*^2^ = 0.39)	Teaching status	1.10	1.05, 1.16	3.70	<0.001
	Bed size	1.00	0.94, 1.05	−0.17	0.867
	RN HPPD^d^	0.98	0.97, 1.00	−2.44	0.015
	Non-RN HPPD	1.03	1.01, 1.05	3.55	<0.001
Surgical (pseudo-*R*^2^ = 0.32)	Teaching status	1.03	0.97, 1.10	1.08	0.278
	Bed size	0.92	0.86, 0.97	−2.83	0.005
	RN HPPD	0.99	0.97, 1.00	−1.62	0.106
	Non-RN HPPD	1.04	1.01, 1.06	2.82	0.005
Rehabilitation (pseudo-*R*^2^ = 0.50)	Teaching status	0.97	0.88, 1.06	−0.72	0.473
	Bed size	1.06	0.96, 1.16	1.15	0.250
	RN HPPD	0.98	0.96, 1.01	−1.17	0.242
	Non-RN HPPD	0.98	0.95, 1.01	−1.42	0.155

^a^Reference group is non-teaching hospitals.

^b^Reference group is hospitals with fewer than 300 beds.

^c^Non-RN HPPD = total non-RN hours divided by total patient days.

^d^RN HPPD = total RN hours divided by total patient days.

There was some tendency for units in larger hospitals to have lower unassisted fall rates. Step-down units in larger hospitals (≥300 beds) were estimated to have 7% (95% CI: 1–12%) lower unassisted fall rates on average than comparable units in smaller hospitals, and surgical unit fall rates were estimated to be 8% (95% CI: 3–14%) lower in larger facilities. For medical–surgical units, the estimated average fall rate was 10% (95% CI: 5–16%) higher in teaching hospitals.

For all unit types except rehabilitation, higher non-RN staffing was associated with higher rates of unassisted falls. Holding other predictors constant, the estimated average fall rate for these unit types was 3–4% higher per additional non-RN HPPD (see Table 2).

**Figure 1:**
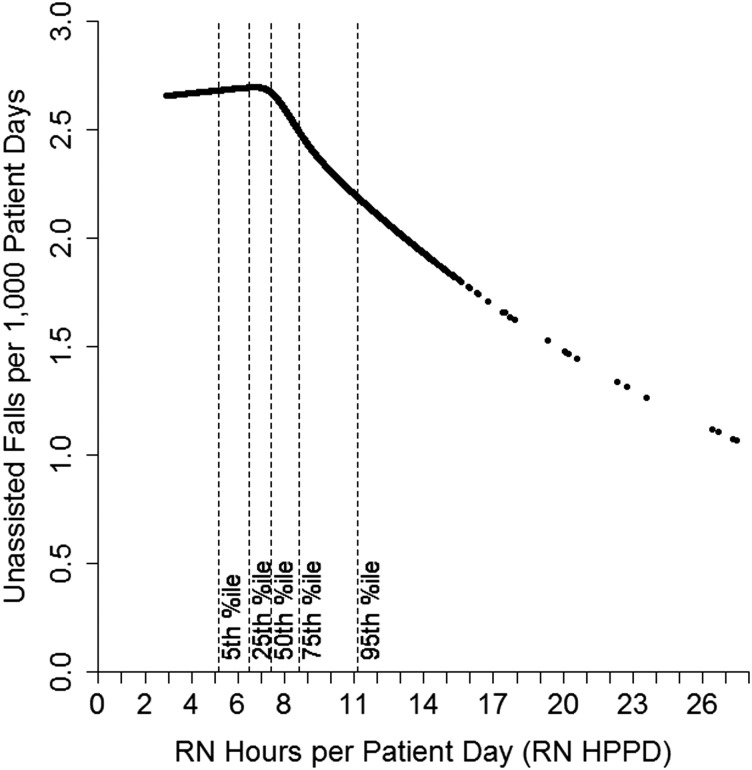
Model-predicted fall rates by RN staffing level for step-down units.

**Figure 2:**
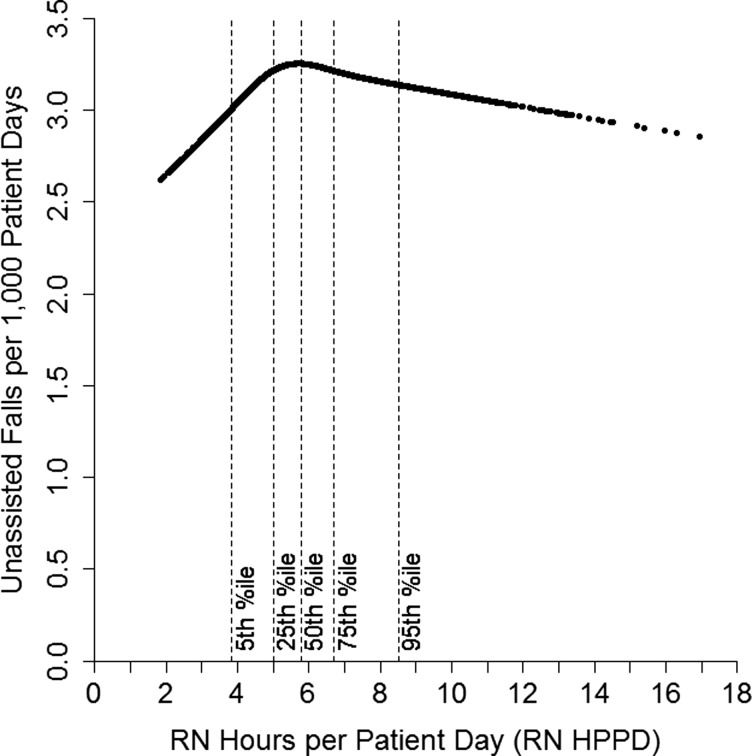
Model-predicted fall rates by RN staffing level for medical units.

There was a significant non-linear association between RN staffing and the unassisted fall rate for step-down and medical units. We explored these associations by plotting the model-predicted rate of unassisted falls per 1000 patient days for an average step-down unit in a small, non-teaching hospital against the values of RN HPPD observed in the study (Fig. 1). An analogous plot for medical units is shown in Fig. 2. In both plots, we set non-RN HPPD to its average for the unit type.

As shown in Fig. 1, there was a weak, positive association between RN staffing and the unassisted fall rate for step-down unit-months with RN HPPD values up to 6.7 (about the 30th percentile). In other words, for lightly staffed units, higher RN staffing levels were associated with slightly higher unassisted fall rates. However, for units staffed at moderate and high levels, higher RN staffing levels were associated with lower fall rates. For a sense of the size of this effect, the predicted unassisted fall rate for a unit-month with staffing at the 95th percentile (11.2 RN HPPD) was 18% lower than for an otherwise equivalent unit-month with median staffing (7.4 RN HPPD).

For medical units at low RN staffing levels, the positive association between RN staffing and the unassisted fall rate was stronger, as shown by the upward slope of the left part of the curve in Fig. 2. At the lowest staffing levels (∼2 RN HPPD), the predicted fall rate was 19% lower than at the median staffing level (5.8 RN HPPD). For unit-months with RN staffing above the median, higher RN staffing was associated with lower fall rates, although the effect was not large. For example, the fall rate at the 95th percentile (8.5 RN HPPD) was only 4% lower than at the median.

There was a linear association between RN staffing and the unassisted fall rate for medical–surgical units. Holding other predictors constant, the estimated average fall rate decreased by 2% (95% CI: 0–3%) per additional RN HPPD. For surgical and rehabilitation units, there was no significant association, linear or non-linear, between RN staffing and the unassisted fall rate.

## Discussion

Our analyses confirm that the association between RN staffing and the rate of unassisted falls varies by unit type. On step-down and medical units, the association between RN staffing and fall rates depended on the level of staffing: at lower staffing levels, the fall rate increased as staffing increased, but at moderate and high staffing levels, the fall rate decreased as staffing increased. Staggs *et al.* [10] reported a similar quadratic association between total nurse staffing and the unassisted fall rate but did not explore differences among unit types in the shape of the quadratic curve; apparently this result was largely driven by the step-down and medical units in the sample.

RN staffing was a not a significant predictor of the unassisted fall rate for surgical and rehabilitation units. On medical–surgical units, RN staffing had a weak inverse association with the unassisted fall rate; that is, higher staffing was associated with lower fall rates.

For all unit types except rehabilitation, non-RN staffing had a positive linear association with the unassisted fall rate; that is, unit-months with higher non-RN staffing tended to have higher rates of unassisted falls. This result is consistent with a previous finding by Lake *et al.* [8] relating higher levels of LPN and nursing assistant staffing with higher total fall rates.

On rehabilitation units, neither staffing variable was associated with the unassisted fall rate. These units serve a distinct patient population, and learning to walk unassisted is a goal of rehabilitation for many patients. Moreover, the presence of other health professionals on rehabilitation units, including physical and occupational therapists, makes it difficult to isolate and assess the effects of nurse staffing levels on the unassisted fall rate.

One limitation of this study is that we could not control for patient acuity (other than by treating each unit type separately) or for patient characteristics such as age. There is some evidence that nursing units with higher average patient acuity tend to have lower total fall rates [16]. This seems plausible, as patients who are too sick to ambulate are at lower risk of falling.

However, if such an association is large enough to be meaningful, and if units with sicker patients tend to have higher levels of RN staffing, we would expect to see an inverse association between RN HPPD and the unassisted fall rate across unit types, even in the absence of any beneficial effect of higher staffing. In other words, if fall rates are driven primarily by patient acuity, we would expect unit-months with higher RN staffing (reflecting sicker patients) to have lower unassisted fall rates. Although we observed this inverse association for at least part of the RN HPPD range for three unit types, there was no such association for the other two unit types. Thus, although patient acuity may have some effect on rates of unassisted falls and would ideally be controlled for, it does not account for the findings of this study.

Another limitation is that we could not take into account the contributions of patient sitters, family members and non-nursing health professionals in preventing falls. Coordination of nursing staff, sitters and family members trained in fall prevention as a method of preventing unassisted falls is a topic for further study.

Causation cannot be inferred from these results. For example, it is clear that unassisted falls were generally more common for unit-months with higher levels of non-RN staffing, but we do not know that higher non-RN staffing causes higher rates of unassisted falls. A third variable, such as the proportion of elderly patients on a unit, may affect both the fall rate and the level of non-RN staffing.

With that caveat stated, higher non-RN staffing does not appear to be effective in preventing unassisted falls. Taken together with the results of Lake *et al.* [8], the findings of this study should give managers pause before increasing non-RN staffing in the hope of reducing falls or fall-related injuries. Nor does the evidence from this study suggest that the unassisted fall rate can be lowered simply by increasing RN staffing without taking into consideration the unit type and its current level of RN staffing.

Clearly, nurse staffing levels are important, but there is a great deal of variation in the unassisted fall rate that cannot be accounted for by RN and non-RN HPPD. Researchers need to look beyond the quantity of nursing care provided for fall prevention solutions; there is more to quality of care than quantity of care. The human capital of nursing staff, including nursing experience, tenure on the unit and education, as well as the ability of unit staff to work as a team, are topics for future research and considerations for managers to keep in mind, along with processes of care and the physical design of the nursing unit.

We limited our focus in this study to the rate of unassisted falls. The proportion of falls assisted is another variable deserving study, and we plan to examine its associations with nurse staffing levels in future research. In addition, we are conducting research to identify factors (e.g. male gender) that increase the odds of a fall being unassisted. Our goal is to contribute to an understanding of falls that will allow hospitals to reduce unassisted fall rates and increase the proportion of falls assisted, thereby reducing the frequency and severity of fall-related injuries to patients.

## Funding

This work was supported by a contract with the American Nurses Association. Funding to pay the Open Access publication charges for this article was provided by the American Nurses Association through a contract with the University of Kansas Medical Center Research Institute.
